# Antineutrophil properties of natural gingerols in models of lupus

**DOI:** 10.1172/jci.insight.138385

**Published:** 2021-02-08

**Authors:** Ramadan A. Ali, Alex A. Gandhi, Lipeng Dai, Julia Weiner, Shanea K. Estes, Srilakshmi Yalavarthi, Kelsey Gockman, Duxin Sun, Jason S. Knight

**Affiliations:** 1 Division of Rheumatology, Department of Internal Medicine, University of Michigan, Ann Arbor, Michigan, USA.; 2 Department of Pharmaceutical Sciences, College of Pharmacy, University of Michigan, Ann Arbor, Michigan, USA.

**Keywords:** Autoimmunity, Inflammation, Lupus, Neutrophils, Phosphodiesterases

## Abstract

Ginger is known to have antiinflammatory and antioxidative effects and has traditionally been used as an herbal supplement in the treatment of various chronic diseases. Here, we report antineutrophil properties of 6-gingerol, the most abundant bioactive compound of ginger root, in models of lupus and antiphospholipid syndrome (APS). Specifically, we demonstrate that 6-gingerol attenuates neutrophil extracellular trap (NET) release in response to lupus- and APS-relevant stimuli through a mechanism that is at least partially dependent on inhibition of phosphodiesterases. At the same time, administration of 6-gingerol to mice reduces NET release in various models of lupus and APS, while also improving other disease-relevant endpoints, such as autoantibody formation and large-vein thrombosis. In summary, this study is the first to our knowledge to demonstrate a protective role for ginger-derived compounds in the context of lupus. Importantly, it provides a potential mechanism for these effects via phosphodiesterase inhibition and attenuation of neutrophil hyperactivity.

## Introduction

Systemic lupus erythematosus (lupus) is the prototypical systemic autoimmune disease, characterized by antinuclear autoantibodies and circulating immune complexes. Lupus has the potential to impact essentially any organ in the body. Antiphospholipid syndrome (APS) is a closely related thromboinflammatory disease defined by the presence of circulating antiphospholipid antibodies (aPL), as detected by anticardiolipin or anti–β-2-glycoprotein I (anti-β_2_GPI) immunoassays or the functional lupus anticoagulant screen (1, 2). APS is a leading acquired cause of thrombosis and pregnancy loss (3) and can occur as a standalone syndrome (primary APS) or in association with lupus (4). In fact, aPL are present in one-third of patients with lupus, amplifying the risk of the thromboembolic events that are among the leading causes of morbidity and mortality in lupus.

Neutrophils release neutrophil extracellular traps (NETs) — tangles of chromatin and microbicidal proteins expelled from neutrophils in response to both infectious and sterile stimuli (5, 6) — which have garnered much recent attention as amplifiers of inflammation and thrombosis in autoimmune diseases such as lupus and APS (7–10). For example, rising levels of blood neutrophils predict glomerulonephritis in lupus (11), while recent studies by our group and others have revealed a key role for neutrophils and NETs in the thrombotic manifestation inherent to lupus and APS. We have shown that patients with APS have higher levels of cell-free DNA and NETs in plasma and that their neutrophils are prone to spontaneous NETosis as compared with healthy controls (12). Furthermore, aPL isolated from patients with APS can trigger healthy control neutrophils to release NETs (12), while also potentiating thrombosis in vivo in neutrophil- and NET-dependent fashion (13). In lupus and APS, NETs promote type I IFN production (14, 15) and autoantibody formation (16, 17). At the same time, lupus-associated autoantibodies, including anti-ribonucleoprotein (anti-RNP) (14), aPL (12), and anti–double-stranded DNA (anti–dsDNA) (18), accelerate NETosis, thereby setting up a vicious cycle.

Ginger has long been perceived to have antiinflammatory and antioxidative properties (19, 20) and has been used traditionally as herbal medicine for the treatment of many ailments, including chronic conditions such as asthma and arthritis. The health-promoting properties of ginger have been attributed to its richness in phenolic phytochemicals (21), such as gingerols and shogaols (22, 23). Of these bioactive compounds, 6-gingerol is the most abundant in fresh ginger, with concentrations of up to 2100 μg/g (24). It has been suggested that ginger may mediate its antiinflammatory effects by reducing levels of proinflammatory cytokines as well as tempering synthesis and secretion of chemokines at sites of inflammation. For example, whole ginger extract can inhibit IL-12, TNF-α, IL-1β, RANTES, and MCP-1 production by LPS-stimulated macrophages (25). Similar effects can be achieved using 6-gingerol alone (26), which also extend to human synoviocytes and chondrocytes (27, 28). In vivo, 6-gingerol blocks activation of NF-κB in phorbol ester–stimulated mouse skin (29) and suppresses the inflammatory response to carrageenan-induced paw edema in rats (30). However, none of these studies have provided a detailed explanation of mechanism.

We were intrigued by recent studies that reported a role for ginger extract and specifically 6-gingerol as inhibitors of cAMP-specific phosphodiesterase (PDE) activity. For example, both ginger extract and 6-gingerol appear to inhibit PDE4 activity in vitro, as tested in human cell lines expressing predominantly the PDE4 isoform (31). In addition, an in silico study identified 3 candidates (including 6-gingerol) from ginger as potential PDE4 inhibitors capable of inhibiting cAMP binding and hydrolysis by PDE4 (32). PDEs (and especially PDE4, the predominant isotype expressed by leukocytes) are attractive therapeutic targets for chronic inflammatory diseases. Theophylline, a nonspecific PDE inhibitor, is leveraged for its antiinflammatory and vasodilatory properties in the treatment of asthma and chronic obstructive pulmonary disease (33). There has also been targeted drug development, including the PDE4 inhibitor apremilast, which is currently used in the clinic in the context of psoriasis, psoriatic arthritis, and Behçet’s disease (34).

The antiinflammatory effects of ginger have yet to be investigated in the context of lupus and APS. Of particular note, no attention has been given to the effects of gingerols on activation and function of neutrophils as thromboinflammatory mediators in lupus and APS. Given evidence that gingerols may similarly exploit cAMP-regulated pathways that we recently characterized in lupus and APS neutrophils (35), here we sought to determine the extent to which ginger-derived compounds might function as natural suppressors of aberrant neutrophil hyperactivity.

## Results

### Gingerols inhibit NETosis elicited by *E. coli* LPS.

**Figure 1:**
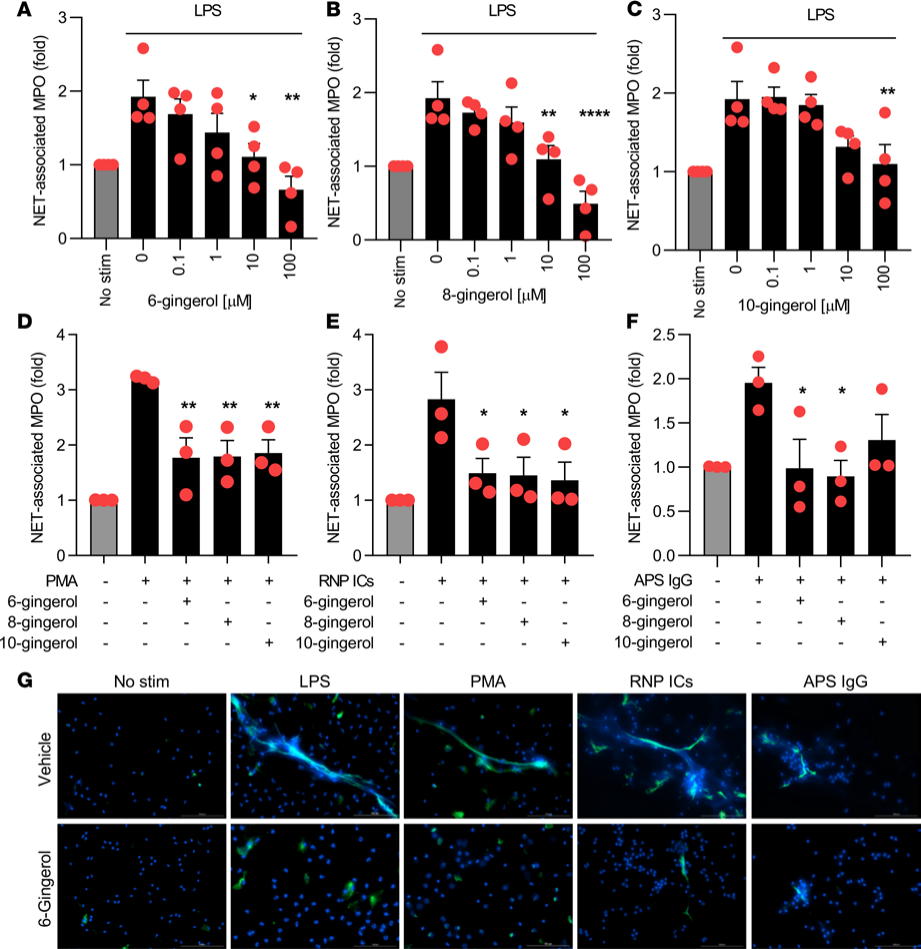
Gingerol suppresses NETosis in response to various stimuli. Human neutrophils were isolated from healthy volunteers and then treated with various stimuli for 3 hours in the presence of different gingerol analogues. NETosis was quantified by measuring the enzymatic activity of nuclease-liberated myeloperoxidase (MPO). Dose response to LPS-mediated NETosis upon treatment with 6-gingerol (**A**), 8-gingerol (**B**), and 10-gingerol (**C**). NETosis in response to PMA (**D**), RNP ICs (**E**), and APS IgG (**F**) was quantified in the presence of 10 μM gingerol. NETosis was assessed by immunofluorescence microscopy (**G**). Neutrophils were treated with LPS, PMA, RNP ICs, or APS IgG in the presence or absence of 6-gingerol (10 μM). Blue, DNA; green, extracellular neutrophil elastase. Scale bar: 100 microns. For **A**–**F**, mean and SEM are presented for *n* = 3 independent experiments; *P < 0.05, **P < 0.01, ****P < 0.0001 as compared with the 0 μM gingerol group by 1-way ANOVA corrected with Dunnett’s test.

We first tested the efficacy of 3 related compounds, 6-gingerol, 8-gingerol, and 10-gingerol (differing only in length of aliphatic side chain), for their ability to suppress NETosis by control neutrophils. We found that both 6- and 8-gingerol at concentrations as low as 10 μM completely neutralized LPS-triggered NETosis (Figure 1, A–C). We then asked whether inhibition would extend to NETosis activated by phorbol 12-myristate 13-acetate (PMA). Indeed, PMA-mediated NETosis was also suppressed by all gingerols (Figure 1D).

### Gingerols inhibit NETosis elicited by lupus and APS autoantibodies.

Neutrophils are activated by various lupus-relevant stimuli, including RNP-containing immune complexes (ICs) and aPL to release NETs. We tested the efficacy of 6-gingerol, 8-gingerol, and 10-gingerol for their ability to suppress NETosis when control neutrophils were activated by either RNP ICs or aPL. All 3 gingerols suppressed RNP IC–induced NETosis at the 10 μM concentration, while 6- and 8-gingerol neutralized aPL-mediated NETosis at the same dose (Figure 1, E and F). The impact of 6-gingerol on NETosis was also assessed by immunofluorescence microscopy with similar results (Figure 1G). The 10 μM concentration was the lowest dose that prevented NETosis in response to LPS (Figure 1A), PMA, and APS IgG (Supplemental Figure 1, A–B; supplemental material available online with this article; https://doi.org/10.1172/jci.insight.138385DS1). At the same time, we found that neutrophils appear healthy over 3 hours, even at concentrations as high as 1 mM 6-gingerol (Supplemental Figure 1C). In summary, these data demonstrate that gingerols have broad anti-NETosis properties that extend to lupus-relevant stimuli, such as RNP ICs and aPL.

### Gingerols inhibit ROS formation by neutrophils.

**Figure 2:**
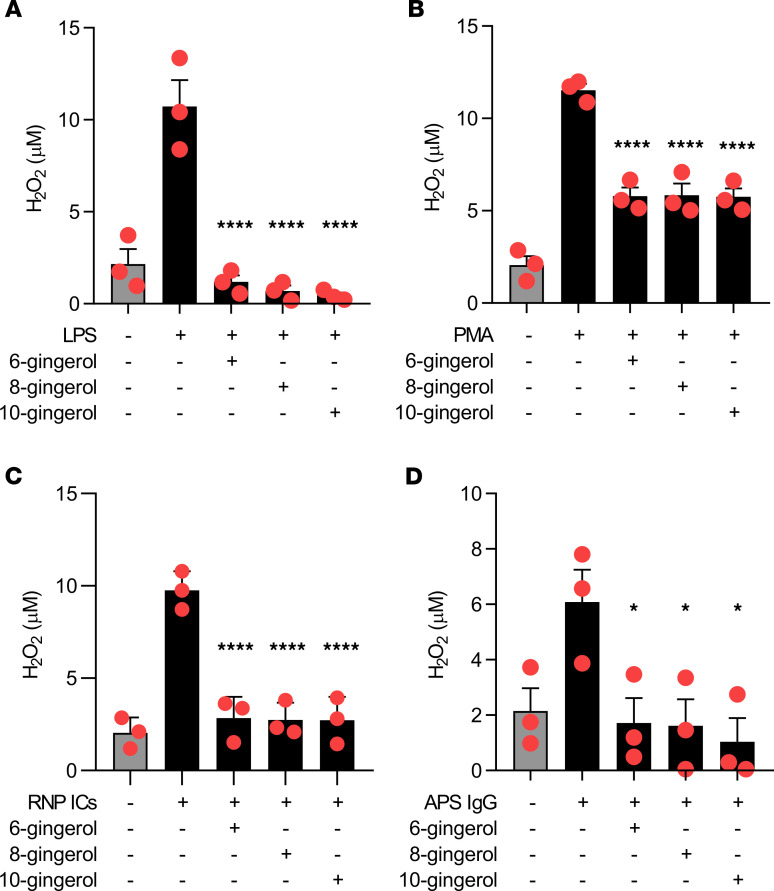
Gingerols suppress ROS. Human neutrophils were treated with various stimuli in the presence of different gingerol analogs for 1 hour. Hydrogen peroxide formation was measured by a colorimetric assay. Mean and SEM are presented for *n* = 3 independent experiments; *P < 0.05, ****P < 0.0001 as compared with the LPS-alone group (**A**), PMA-alone group (**B**), RNP ICs–alone group (**C**), or APS IgG–alone group (**D**) by 1-way ANOVA corrected with Dunnett’s test.

Ginger has been reported to have antioxidative properties. Thus, we reasoned that gingerols might suppress NETosis by preventing the neutrophil oxidative burst, as ROS are required for most forms of NETosis. All gingerols suppressed formation of H_2_O_2_ in neutrophils, whether stimulated by LPS, PMA, RNP ICs, or aPL (Figure 2). Taken together, these data suggest a potential mechanism by which gingerols mitigate NETosis, namely by suppressing ROS formation.

### 6-Gingerol inhibits cAMP-specific PDE activity.

**Figure 3:**
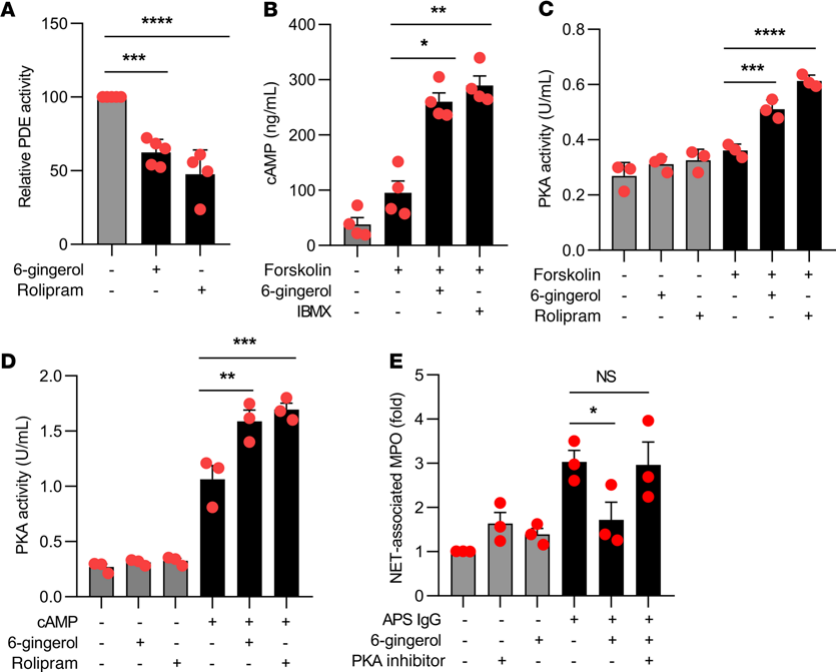
6-Gingerol blocks PDE activity and raises cAMP levels. Human neutrophils were treated with 6-gingerol. Some samples were additionally treated with forskolin, cAMP, and synthetic PDE4 inhibitors (rolipram and IBMX) as indicated. PDE activity (**A**), cAMP levels (**B**), and PKA activity (**C** and **D**) were measured with kits as described in Methods. In **E**, neutrophils were treated with APS IgG in the presence or absence of 6-gingerol and/or PKA inhibitor. NETosis was quantified by measuring the enzymatic activity of nuclease-liberated myeloperoxidase (MPO). Mean and SEM are presented for *n* = 3–4 independent experiments; *P < 0.05, **P < 0.01, ***P < 0.001, ****P < 0.0001 by 1-way ANOVA corrected with Dunnett’s test.

Ginger extracts and specifically 6-gingerol have been suggested to function as PDE inhibitors. Here, we reasoned that 6-gingerol might suppress NETosis through modulation of cAMP levels and downstream pathways. We first tested the effect of 6-gingerol on PDE activity in neutrophils. We found that 6-gingerol reduced PDE activity by 40%, as compared with a 50% reduction by the synthetic PDE4 inhibitor rolipram (Figure 3A). We also measured intracellular concentrations of cAMP upon stimulation of neutrophils with the adenylate cyclase activator forskolin. Interestingly, both 6-gingerol and 3-isobutyl-1-methylxanthine (IBMX) (another synthetic PDE inhibitor) significantly potentiated intracellular cAMP concentrations, as compared with untreated samples (Figure 3B). Having documented a gingerol-mediated increase in intracellular concentrations of cAMP, we considered that activity of the key downstream cAMP-dependent kinase, PKA, might also increase in neutrophils. Indeed, 6-gingerol significantly enhanced neutrophil PKA activity (Figure 3, C and D). Furthermore, the suppressive effects of 6-gingerol on NETosis could be mitigated by blocking PKA activity (Figure 3E). In summary, these data demonstrate that 6-gingerol attenuates NETosis in vitro through a mechanism that at least partially depends on inhibition of PDE activity, potentiation of cAMP levels, and resultant activation of PKA.

### 6-Gingerol attenuates lupus-relevant disease activity in mice.

**Figure 4:**
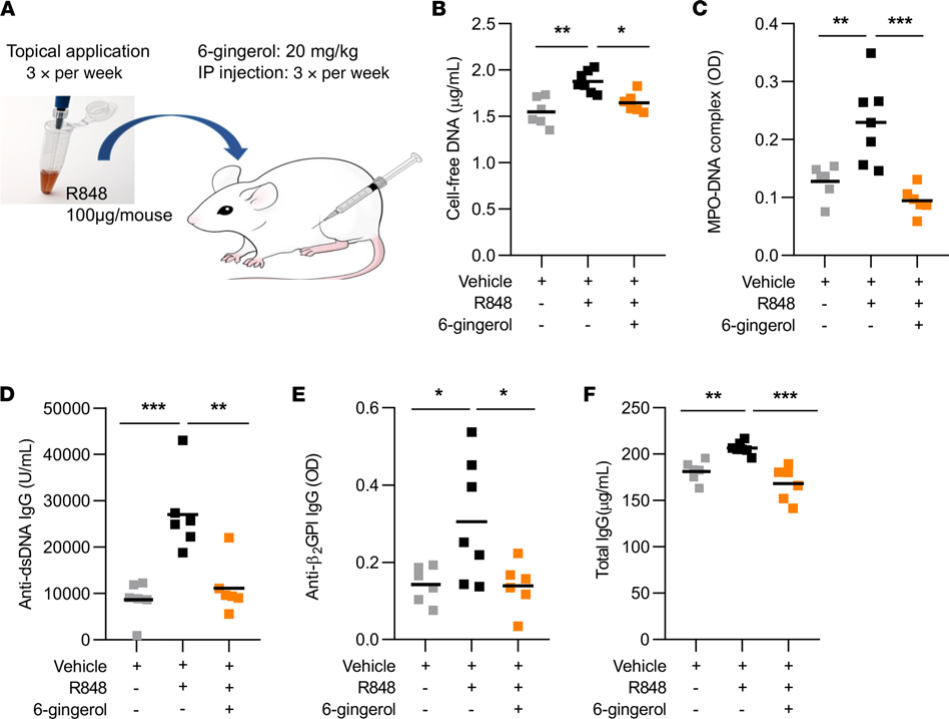
6-Gingerol attenuates NET release and autoantibody formation in a lupus mouse model. BALB/c mice were treated topically with TLR7 agonist (R848) or vehicle DMSO for 6 weeks (3 times per week). Some mice were additionally injected (i.p.) with 20 mg/kg 6-gingerol (3 times per week). Schematic of the TLR7 agonist–induced (R848) lupus model (**A**). NET levels in serum were assessed by measuring cell-free DNA (**B**) and MPO-DNA complexes (**C**). Anti–double-stranded DNA (anti-dsDNA) (**D**), anti–β-2 glycoprotein I (β_2_GPI) IgG (**E**), and total IgG (**F**) levels in serum were assessed by ELISA. Mean is presented as a horizontal line; *P < 0.05, **P < 0.01, ***P < 0.001 as compared with the R848-alone group by 1-way ANOVA corrected with Dunnett’s test.

**Figure 5:**
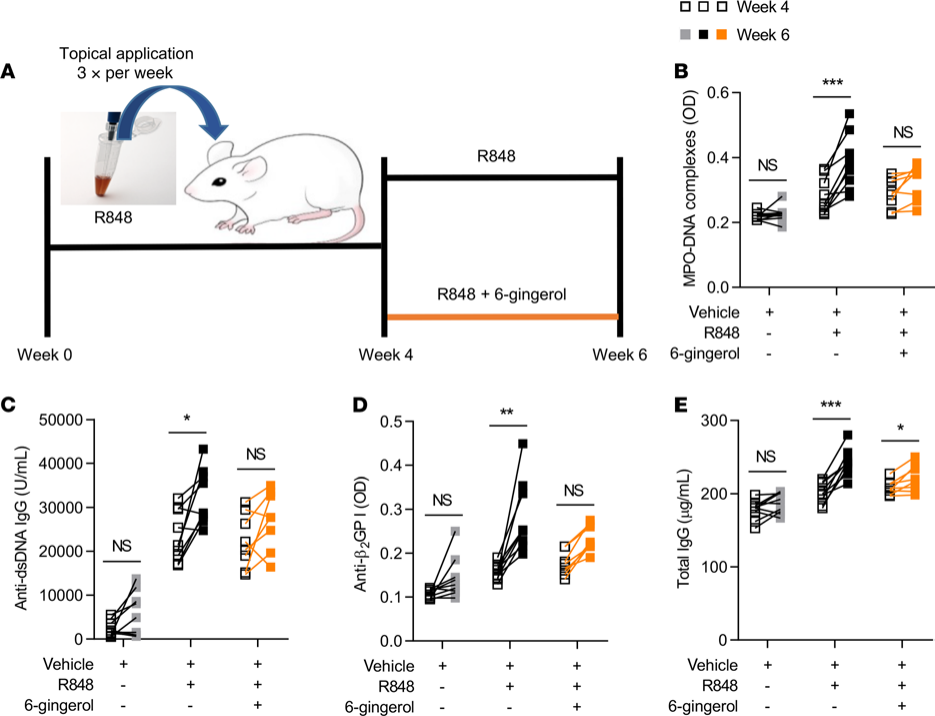
Efficacy of 6-gingerol treatment after development of lupus phenotype in a lupus mouse model. BALB/c mice were treated topically with TLR7 agonist (R848) or vehicle DMSO for 6 weeks (3 times per week). Starting at week 4 of treatment, some mice were additionally injected (i.p.) with 20 mg/kg 6-gingerol (3 times per week). Schematic of the TLR7 agonist–induced (R848) lupus model by week 4 followed by 6-gingerol treatment (**A**). NET levels in serum were assessed before and after 6-gingerol treatment by measuring MPO-DNA complexes (**B**). Anti–double-stranded DNA (anti-dsDNA) (**C**), anti–β-2 glycoprotein I (β_2_GPI) IgG (**D**), and total IgG (**E**) levels in serum were assessed by ELISA before and after 6-gingerol treatment. Mean is presented as a horizontal line; *P < 0.05, **P < 0.01, ***P < 0.001 by paired *t* test.

We next tested the efficacy of 6-gingerol on disease activity in a lupus mouse model. Administration of 6-gingerol to TLR7 agonist–treated mice (Figure 4A) resulted in a marked reduction in plasma NET levels, as evident by reductions in both cell-free DNA and myeloperoxidase-DNA (MPO-DNA) complexes (Figure 4, B and C). Key antibodies, including anti-dsDNA, anti-β_2_GPI, and total IgG, were also reduced (Figure 4, D–F). Spleen size, total leukocytes, lymphocytes, neutrophils, and platelets were not significantly altered by 6-gingerol (Supplemental Figure 2, A–E). Given that 6-gingerol inhibited cAMP-specific PDE activity in vitro, we reasoned that it would also increase cAMP concentration in vivo and thereby inhibit proinflammatory cytokines — similar to synthetic PDE inhibitors (36). As expected, 6-gingerol–treated mice showed significantly lower levels of both IFN-γ and TNF-α (Supplemental Figure 3, A–B). We next investigated the efficacy of 6-gingerol treatment after disease onset in the same lupus model. To do this, 6-gingerol treatment was delayed for 4 weeks relative to the initiation of the TLR7 agonist; both TLR7 agonist and 6-gingerol were then administered together for the final 2 weeks of the experiment (Figure 5A). While vehicle-treated mice accrued higher levels of NETs and autoantibodies between weeks 4 and 6, these increases were markedly blunted in the mice receiving 6-gingerol (Figure 5, B–E, and Supplemental Figure 4, A–D). Taken together, these data suggest that 6-gingerol reduces lupus-relevant NET release and autoantibody formation in vivo.

### 6-Gingerol attenuates aPL-mediated venous thrombosis.

**Figure 6:**
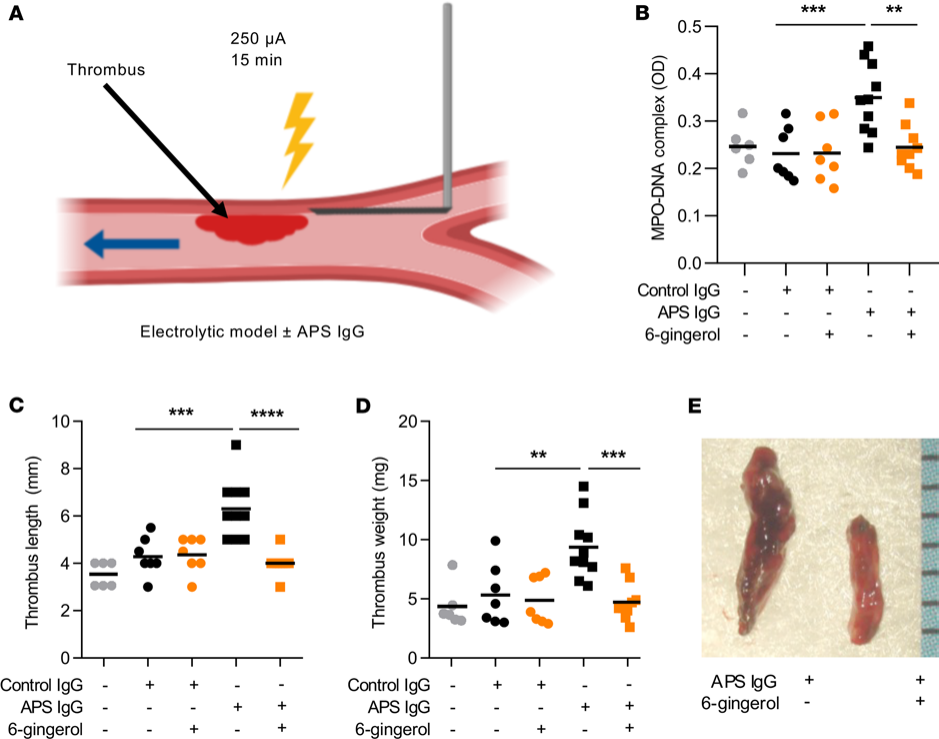
6-Gingerol prevents aPL-mediated acceleration of venous thrombosis. Schematic of the electrolytic model of venous thrombosis (**A**). Direct current results in the release of free radicals within the inferior vena cava, which activate endothelial cells and initiate a thrombogenic environment in the presence of constant blood flow. MPO-DNA complexes were measured in serum of mice treated with control IgG or APS IgG in the presence or absence of 6-gingerol (**B**). Thrombus formation was assessed at 24 hours. Thrombus length (**C**) and thrombus weight (**D**) were measured. Representative thrombi (**E**). **P < 0.01, ***P < 0.001, ****P < 0.0001 by 1-way ANOVA corrected with Dunnett’s test.

**Figure 7:**
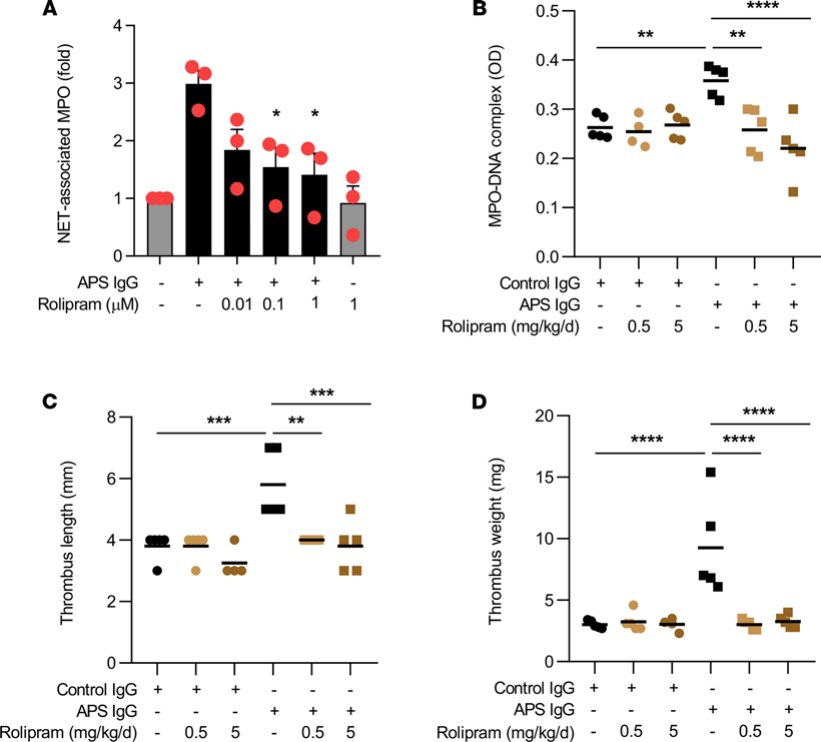
The synthetic PDE4 inhibitor rolipram suppresses APS-mediated NETosis and venous thrombosis. Human neutrophils were stimulated with APS IgG for 3 hours. Some samples were additionally treated with the PDE4 inhibitor rolipram. NETosis was quantified by measuring the enzymatic activity of nuclease-liberated myeloperoxidase (MPO) (**A**). MPO-DNA complexes were assessed for control IgG- or APS IgG-treated mice in the presence or absence of rolipram (**B**). Thrombus formation was assessed at 24 hours. Thrombus length (**C**) and thrombus weight (**D**) were measured; **P < 0.01, ***P < 0.001, ****P < 0.0001 by 1-way ANOVA corrected with Dunnett’s test.

The pathologic role of neutrophils and NETs in thrombosis has been observed in both human (37–39) and mouse studies (13, 40, 41). Since 6-gingerol suppressed aPL-mediated NETosis in vitro, we reasoned that it might also mitigate aPL-accelerated NETosis and thrombosis in vivo. We induced large-vein thrombosis utilizing an electrolytic inferior vena cava (IVC) model that we have described previously (35, 42) (Figure 6A). Administration of aPL increased serum NET levels, which returned to baseline when mice were treated with 6-gingerol (Figure 6B). As expected, administration of aPL, but not control IgG, increased thrombus length and weight, which again returned to control levels upon administration of 6-gingerol (Figure 6, C–E). We also examined the effect of the synthetic PDE4 inhibitor rolipram on NETosis and venous thrombosis. Similar to 6-gingerol, rolipram suppressed aPL-mediated NETosis in vitro in dose-dependent fashion (Figure 7A). In the aforementioned model of aPL-accelerated large-vein thrombosis, administration of rolipram to the APS mice returned circulating MPO-DNA complexes (Figure 7B) and thrombus size (Figure 7, C and D) to levels seen in control mice. In summary, these data demonstrate that 6-gingerol suppresses NETosis and venous thrombosis in vivo and provide further support of the idea that 6-gingerol functions as a PDE4 inhibitor, given that its effects are phenocopied by a synthetic PDE4 inhibitor.

### Kinetics of 6-gingerol in plasma and neutrophils.

**Figure 8:**
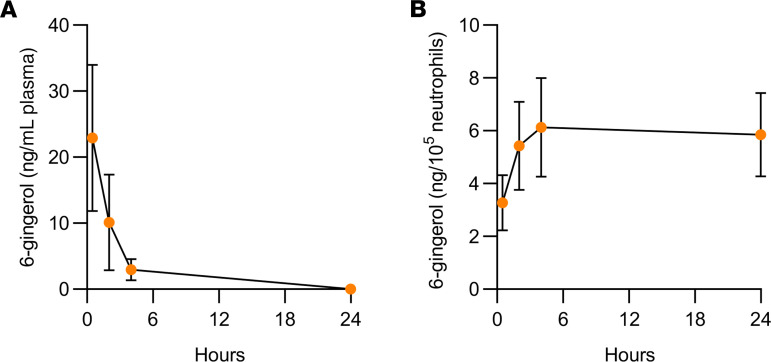
Kinetics of 6-gingerol in plasma and neutrophils following injection (i.p.) with 20 mg/kg 6-gingerol. Male C57BL/6 mice were injected (i.p.) with 20 mg/kg 6-gingerol, and peripheral blood was collected after 0.5, 2, 4, and 24 hours. 6-Gingerol concentrations in plasma (**A**) and in neutrophils (**B**) were then quantitated. Values and error bars represent mean ± SEM, respectively.

Finally, we preformed pharmacokinetic studies of 6-gingerol. In particular, we focused on the distribution of 6-gingerol in neutrophils, the major theme of the above experiments. Interestingly, we observed accumulation of 6-gingerol in neutrophils at the same time levels were dropping in plasma (Figure 8). These results are in line with previous work suggesting that ginger biophenolics are rapidly cleared from plasma by conversion into glucuronide conjugates but then reconverted into their free forms in tissues by enzymes such as β-glucuronidases (43).

## Discussion

Here, we have revealed antineutrophil properties of 6-gingerol that may have protective effects in disease states, such as lupus and APS. In vivo, we characterized 2 lupus-relevant inflammatory models and found that 6-gingerol reduced NETosis in both. Beyond inhibition of NETosis, we also saw positive effects of disease phenotypes, such as autoantibody formation and thrombosis, and observed that 6-gingerol behaved very similarly to a synthetic PDE4 inhibitor.

Mechanistically, we found that the effects of 6-gingerol on neutrophils are at least partially attributable to its ability to inhibit PDE activity (Supplemental Figure 5). In vitro data demonstrate that 6-gingerol increases intracellular concentrations of cAMP and enhances PKA activity in neutrophils. Furthermore, 6-gingerol suppressed the production of proinflammatory cytokines, such as TNF-α and IFN-γ, similar to PDE4 inhibitors (36), therefore exerting an overall antiinflammatory effect in TLR7 agonist–induced lupus. The activation of cAMP/PKA by 6-gingerol would seem to support this pathway as a potential therapeutic target in lupus and APS with drugs such as PDE4 inhibitors (44). This is in agreement with our previous work demonstrating the potential role of adenosine receptors, cAMP, and PKA in suppressing APS-mediated NETosis and thrombosis (35).

Ginger has previously been reported to have antiinflammatory (45), antioxidant (46), and antithrombotic (47) effects. Regarding the latter, gingerol-related compounds, including 6-gingerol, have been shown to prevent platelet aggregation (48), although this finding has not been reproduced by all groups (49). Here, it is certainly possible that both antiplatelet and antineutrophil properties are contributing to protection against venous thrombosis. Beyond thrombosis, ginger has also been considered as a treatment for rheumatic diseases. Several clinical studies have suggested beneficial effects of ginger for the treatment of arthritis. For example, ginger extract was found to alleviate pain and decrease joint swelling (50, 51); to our knowledge, neutrophil phenotypes have not been considered in any of these clinical studies. In mice, there is evidence that ginger may modulate inflammatory cell trafficking, with decreased recruitment of neutrophils, eosinophils, and monocytes into inflamed airways (52). Here, there was a trend toward reduced neutrophil numbers in circulation (Supplemental Figure 2A), and future studies should endeavor to look specifically at the effect of 6-gingerol on neutrophil trafficking in models of autoimmunity.

We found that 6-gingerol concentrations as low as 10 μM were effective in mitigating NETosis. This is a concentration that provides no cellular toxicity over 24 hours (53). In fact, for the duration of our assay (3 hours), we did not detect toxic effects, even at molar concentration of 6-gingerol. Ginger extracts are generally regarded as safe (54), with 2 grams daily (approximately 25 mg/kg) demonstrating low levels of toxicity and high levels of tolerability in humans (55). Given the faster metabolic rate of mice as compared with humans (and the allometric scaling factor of 10), 250 mg/kg ginger extract has been a common dose for murine in vivo studies. As ginger extracts typically consist of 5% active phenolic compounds (mostly 6-gingerol), the dose chosen here (10 mg/kg 6-gingerol) lines up well with previous work.

Free forms of ginger biophenolics, including 6-gingerol, can be detected in human plasma upon oral administration of ginger extracts (56, 57). These compounds are rapidly cleared from plasma and converted into glucuronide conjugates. A recent study proposed a model whereby these conjugated forms are reconverted into their free forms in tissues by enzymes, such as β-glucuronides (43), which are known to be abundantly expressed by neutrophils (58). Consistent with this model, we appreciated accumulation of 6-gingerol in neutrophils at the same time levels were dropping in plasma.

While it is unlikely that ginger extract or 6-gingerol would find a role as a primary therapeutic in individuals with active disease, one wonders if future studies might administer ginger supplements to individuals at high risk for autoimmune conditions and/or cardiovascular disease (for example, individuals with autoantibodies who have yet to have clinical events or patients with cardiovascular risk factors). In such scenarios, its antineutrophil properties might prove protective against disease emergence. Based on the data presented here, we would argue that such studies should have not only clinical endpoints, but also mechanistic endpoints focusing on neutrophil activity.

## Methods

### Purification of patient IgG.

IgG was purified from APS or control sera with a Protein G Agarose Kit following the manufacturer’s instructions (Pierce) as previously described (35). Briefly, serum was diluted in IgG binding buffer and passed through a Protein G Agarose column at least 5 times. IgG was then eluted with 0.1 M glycine and neutralized with 1 M Tris. This was followed by overnight dialysis against PBS at 4°C. IgG purity was verified with Coomassie staining, and concentrations were determined by BCA protein assay (Pierce) according to the manufacturer’s instructions. All IgG samples were determined to be free of detectable endotoxin by the Pierce LAL Chromogenic Endotoxin Quantitation Kit (88282) according to the manufacturer’s instructions.

### Human neutrophil purification and NETosis assays.

Blood from healthy volunteers was collected into heparin tubes by standard phlebotomy techniques. The anticoagulated blood was then fractionated by density-gradient centrifugation using Ficoll-Paque Plus (GE Healthcare). Neutrophils were further purified by dextran sedimentation of the red blood cell layer, before lysing residual red blood cells with 0.2% sodium chloride. Neutrophil preparations were at least 95% pure, as confirmed by both flow cytometry and nuclear morphology.

To assess NETosis, neutrophils were resuspended in RPMI media (Gibco) supplemented with 0.5% BSA (MilliporeSigma) and 0.5% fetal bovine serum (Gibco), which had been heat-inactivated at 56°C. Neutrophils (1 × 10^5^/well) were then cultured in 96-well plates at 37°C with 100 nM PMA (MilliporeSigma), 2 μg/mL LPS (*Escherichia coli* O26:B6, L2654, MilliporeSigma), 10 μg/mL APS IgG, or 10 μg/mL RNP ICs. RNP ICs were formed by mixing IgG purified from 3 individuals with lupus and anti-RNP positivity with SmRNP (Arotec). In some cases, cultures were also supplemented with 6-gingerol, 8-gingerol, and 10-gingerol (Cayman Chemical). After 3 hours in culture, NET-associated MPO activity was measured as follows. The culture media was discarded (to remove any soluble MPO) and replaced with 100 μL RPMI supplemented with 10 U/mL Micrococcal nuclease (Thermo Fischer Scientific). After 10 minutes at 37°C, digestion of NETs was stopped with 10 mM EDTA. Supernatants were transferred to a V-shaped 96-well plate and centrifuged at 350*g* for 5 minutes to remove debris. Supernatants were then transferred into a new plate. To measure for MPO activity, an equal volume of 3,3′,5,5′-tetramethylbenzidine (3,3′,5,5′-TMB) substrate (1 mg/mL, Thermo Fischer Scientific) was added to each well. After 10 minutes of incubation in the dark, the reaction was stopped by the addition of 50 μL of 1 mM sulfuric acid. Absorbance was measured at 450 nm using a Cytation 5 Cell Imaging Multi-Mode Reader.

### Immunofluorescence microscopy.

For immunofluorescence microscopy, 1.5 × 10^5^ neutrophils were seeded onto coverslips coated with 0.001% poly-l-lysine (MilliporeSigma) and fixed with 4% paraformaldehyde for 15 minutes at room temperature. Blocking was with 1% BSA overnight at 4°C. The primary antibody was against neutrophil elastase (Abcam, 21595, diluted 1:100), and the FITC-conjugated secondary antibody was from Southern Biotech (4052-02, diluted 1:250). DNA was stained with Hoechst 33342 (Invitrogen). Images were collected with a Cytation 5 Cell Imaging Multi-Mode Reader.

### H_2_O_2_ assay.

The generation of H_2_O_2_ was quantified as described previously (17). Briefly, H_2_O_2_ production was detected by a colorimetric assay, with 50 μM Amplex Red reagent (Invitrogen), and 10 U/ml horseradish peroxidase (MilliporeSigma) was added to the culture medium. Absorbance was measured at 560 nm and linearity was assured with an H_2_O_2_ standard curve.

### Measurement of PDE activity.

Human neutrophils (1 × 10^7^) were washed twice with cold PBS and pelleted by centrifugation at 2500*g* for 5 minutes. The cell pellet was resuspended in 100 μL RIPA buffer (MilliporeSigma, R0278) supplemented with protease inhibitors (Roche Diagnostics GmbH, 35440400) for 15 minutes on ice. The mixture was centrifuged at 14,000*g* for 5 minutes to clear cell debris. The activity of PDE was measured using the Bridge-It cAMP-PDE assay kit (Mediomics, catalog PD-1016). The supernatant was mixed with the reaction mixture according to the manufacturer’s instructions in the presence or absence of 10 μM 6-gingerol or 0.1 μM of the PDE4 inhibitor rolipram and allowed to proceed for 1 hour at 37°C. The reaction was stopped and assay solution was added. After 30 minutes at 37°C, fluorescence was measured with a Synergy HT Multi-Mode Microplate Reader (BioTek) at excitation 480 nm and emission. Relative activity was calculated and normalized to mean control values.

### Measurement of intracellular cAMP.

cAMP levels in human neutrophils were measured using the Bridge-It cAMP Designer fluorescence assay kit (Mediomics, catalog 122934). Briefly, neutrophils (1 × 10^5^) were washed twice with PBS and resuspended in 100 μL Krebs-Ringer Bicarbonate Buffer (without IBMX). To investigate the effect of 6-gingerol on the cAMP level, neutrophils were incubated at room temperature for 30 minutes in the presence or absence of 10 μM 6-gingerol. Neutrophils were then stimulated with 100 μM forskolin for 10 minutes. Samples were centrifuged at 12,000*g* for 2 minutes and supernatants were discarded. The cAMP designer assay solution was then added to the cell pellet and carefully transferred to 96-well black-side clear-bottom plates. The plate was incubated at room temperature for 30 minutes before measuring fluorescence with a Synergy HT Multi-Mode Microplate Reader (BioTek) at excitation 480 nm with a band pass filter of 20 nm and emission 540 nm with a band pass filter of 40 nm.

### Measurement of PKA activity.

Neutrophils (1 × 10^7^) were preincubated with 10 μM 6-gingerol or 0.1 μM of the PDE4 inhibitor rolipram and then treated with 100 μM forskolin or 1 μM cAMP. The activity of PKA was then measured using the PKA activity assay kit (Arbor Assays, catalog K027-H1). Briefly, neutrophils were washed twice with cold PBS and pelleted by centrifugation at 2500*g* for 5 minutes. The cell pellet was resuspended in 1 mL activated cell lysis buffer for 30 minutes on ice. The mixture was centrifuged at 10,600*g* at 4°C for 10 minutes to pellet the cell debris. In a 96-well plate, the supernatant was mixed with the kinase assay buffer, followed by addition of ATP, and the plate was incubated at 30°C for 90 minutes. The plate was washed 4 times, followed by the addition of donkey anti-rabbit IgG HRP conjugate and the rabbit phosphor PKA substrate antibody, and then incubated at room temperature for 60 minutes. The plate was washed 4 times, followed by the addition of TMB substrate and incubation at room temperature for 30 minutes. Stop solution was added and the optical density was read at 450 nm using a Cytation 5 Cell Imaging Multi-Mode Reader.

### Animal housing and treatments.

Mice were purchased from The Jackson Laboratory, housed in a specific pathogen–free barrier facility, and fed standard chow. Male C57BL/6 mice were used for venous thrombosis experiments, and 6-gingerol (AdooQ Bioscience) was administered by intraperitoneal injection, 10 mg/kg, daily. Therapy was always started 1 day before IVC surgery and continued through the duration of the experiment. Female BALB/c mice were used for resiquimod-induced (R848-induced) lupus, and 6-gingerol was administered by intraperitoneal injection at 20 mg/kg 3 times per week.

### In vivo venous thrombosis.

To model large-vein thrombosis, we employed an electrolytic model that has been used previously by our group and others (35, 42). Briefly, after exposure of the IVC, any lateral branches were ligated using 7-0 Prolene suture (back branches remained patent). A 30-gauge silver-coated copper wire (KY-30-1-GRN, Electrospec) with exposed copper wire at the end was placed inside a 25-gauge needle and inserted into the IVC, where it was positioned against the anterior wall and functioned as the anode. Another needle was implanted subcutaneously, completing the circuit (cathode). A constant current of 250 μA was applied for 15 minutes. The current was supplied by a voltage-to-current converter as described in detail previously (42). After removal of the needle, the abdomen was closed. Before recovery from anesthesia, mice received a single intravenous injection of either control or APS IgG (500 μg). Twenty-four hours later, mice were humanely euthanized, blood was collected, and thrombus size was measured.

### Quantification of MPO-DNA complexes.

MPO-DNA complexes were quantified similarly as previously described (59). This protocol used several reagents from the Cell Death Detection ELISA kit (Roche). First, a high-binding EIA/RIA 96-well plate (Costar) was coated overnight at 4°C with anti-human MPO antibody (Bio-Rad0400-0002) and diluted to a concentration of 0.5 μg/mL in coating buffer (Cell Death Detection ELISA kit). The plate was washed 3 times with wash buffer (0.05% Tween 20 in PBS) and then blocked with 1% BSA in PBS for 1 hour at room temperature. The plate was again washed 3 times, before incubating for 1 hour at room temperature with 1:500 mouse serum in the aforementioned blocking buffer. The plate was washed 5 times and then incubated for 1 hour at room temperature with 1× anti-DNA antibody (HRP-conjugated; Cell Death Detection ELISA kit) diluted 1:100 in blocking buffer. After 5 more washes, the plate was developed with 3,3′,5,5′-TMB substrate (Invitrogen), followed by a 2 N sulfuric acid stop solution. Absorbance was measured at a wavelength of 450 nm with a Synergy HT Multi-Mode Microplate Reader (BioTek). Data were normalized to an in vitro–prepared NET standard included on every plate.

### R848 treatment.

Female BALB/c mice were treated with the TLR7 agonist R848 (Enzo Life Science) as previously described (60) with slight modifications. Epicutaneous application was to the ear 3 times per week, with 100 μg R848 dissolved in 8 μL DMSO. For 6-gingerol treatment, some mice were injected intraperitoneally with 20 μg/kg 6-gingerol on the same days as R848 treatment. Serum and tissues were collected after 6 weeks of treatment.

### Quantification of anti-dsDNA and total IgG.

Kits for mouse anti-dsDNA and mouse total IgG were purchased from Alpha Diagnostic International (catalog 5120 and 6320, respectively) and performed according to the manufacturer’s instructions.

### Quantification of anti-β_2_GPI.

High-binding EIA/RIA plates were coated overnight at 4°C with 1 μg/mL mouse β_2_GPI (R&D 6575-AH) diluted in coating buffer from a Cell Death Detection ELISA kit. Plates were then washed with 0.05% Tween 20 in PBS and blocked with 1% BSA in PBS for 1 hour at room temperature. The plate was again washed, before incubation for 1 hour at room temperature with 1:500 mouse serum in the aforementioned blocking buffer. The plate was washed and then incubated for 1 hour at room temperature with anti-mouse IgG HRP (Jackson ImmunoResearch, 115-035-068) diluted 1:20,000 in blocking buffer. The plate was washed and developed with 3,3′,5,5′-TMB substrate (Invitrogen) followed by a 2 N sulfuric acid stop solution. Absorbance was measured at a wavelength of 450 nm with a Synergy HT Multi-Mode Microplate Reader (BioTek).

### Quantification of cell-free DNA.

Cell-free DNA was quantified in mouse serum using a Quant-iT PicoGreen dsDNA assay kit (Invitrogen) according to the manufacturer’s instructions.

### Complete blood counts.

Peripheral leukocyte and platelet counts were determined with an automated Hemavet 950 counter (Drew Scientific).

### Quantification of IFN-γ and TNF-α.

Kits for quantitative detection of mouse IFN-γ (88-7314) and TNF-α (88-7324) were purchased from Invitrogen and performed according to the manufacturer’s instructions.

### Pharmacokinetic studies of 6-gingerol in neutrophils.

Male C57BL/6 mice were treated with 20 mg/kg 6-gingerol by intraperitoneal injection. Peripheral blood was collected 0.5, 2, 4, and 24 hours after 6-gingerol treatment. Plasma was collected and neutrophils were isolated from peripheral blood using the EasySep negative selection kit (STEMCELL Technologies, catalog 19762) according to the manufacturer’s instructions.

### Bioanalysis.

Neutrophil samples were suspended with 100 μL of 20% acetonitrile and then subjected to 3 freeze-thaw cycles. Samples were sonicated twice (3 seconds each) to breakdown neutrophil pellets, with cooling on ice for 15 seconds between sonications. A stock solution of 6-gingerol was diluted to 0.25, 0.5, 1, 2.5, 5, 10, 25, 50, and 100 ng/mL methanol to prepare working solutions. To prepare calibration curves, 30 μL blank cell suspension was spiked with 30 μL of the various working solutions above respectively; whereas 30 μL of tested samples was spiked with 30 μL methanol. All samples, were spiked with 30 μL internal standard solution. All the samples were vortexed for extraction and centrifuged at 10,000*g* at 4°C for 15 minutes. Finally, isolated supernatants from each tube were submitted for subsequent LC-MS analysis. Plasma samples were prepared in a similar way.

### LC-MS analysis.

The mass spectrometer AB Sciex QTRAP 5500, coupled with the Shimazu LC20A liquid chromatography system, was operated in multiple-reaction monitoring–positive mode for determination of 6-gingerol in neutrophil pellets and plasma samples. Chromatographic separation was accomplished with the application of a Waters XBridge C18, 2.1 × 50 mm, 5 μm column. The mobile phase A consisted of 0.1% formic acid in deionized water, and mobile phase B consisted of 100% acetonitrile supplemented with 0.1% formic acid. The HPLC was subjected to gradient elution, at 0.4 mL/minute, and positive ion mode was adopted in the mass spectrometer. The mobile phase B was maintained at 1% during the initial 0.5 minutes and then increased to 99% from 0.5 minutes to 2.5 minutes. The composition of mobile phase B was decreased to 1% at 4.5 minutes and maintained there until the end of the run time at 6.6 minutes. Mass/charge (*m/z*) transitions 295.1**→**137.2 and 295.1**→**177.0 were monitored for 6-gingerol quantification in biological samples, while *m/z* transition 455.2**→**425.2 was monitored for internal standard.

### Statistics.

Data were analyzed with GraphPad Prism software (version 8). For continuous variables, group means were compared by either 2-tailed *t* test (2 groups) or 1-way ANOVA (more than 2 groups); correction for multiple comparisons was by Dunnett’s test. Statistical significance was defined as *P* < 0.05.

### Study approval.

This study complied with all relevant ethical regulations and was approved by the University of Michigan Institutional Review Board. All participants provided informed consent for blood donation. Mouse experimental protocols were approved by the University of Michigan Institutional Animal Care and Use Committee, and all relevant ethical regulations were followed.

## Author contributions

RAA, AAG, LD, JW, SKE, SY, KG, and DS conducted experiments and analyzed data. RAA and JSK designed the study and analyzed data. All authors drafted the manuscript and gave approval before submission.

## Supplementary Material

Supplemental data

## Version 1 12/29/2020

In-Press Preview

## Version 2 02/08/2021

Electronic publication
