# Supplementary material for: Antineutrophil properties of natural gingerols in models of lupus
Source: JCI Insight. 2021 Feb 8;6(3):e138385. doi: 10.1172/jci.insight.138385 (PMC7934838; doi:10.1172/jci.insight.138385)
Supplement: Supplemental data [file jciinsight-6-138385-s114.pdf]

SUPPLEMENTAL MATERIAL

Antineutrophil properties of natural gingerols in models of lupus

Ali *et al.*

Supplemental Figure 1

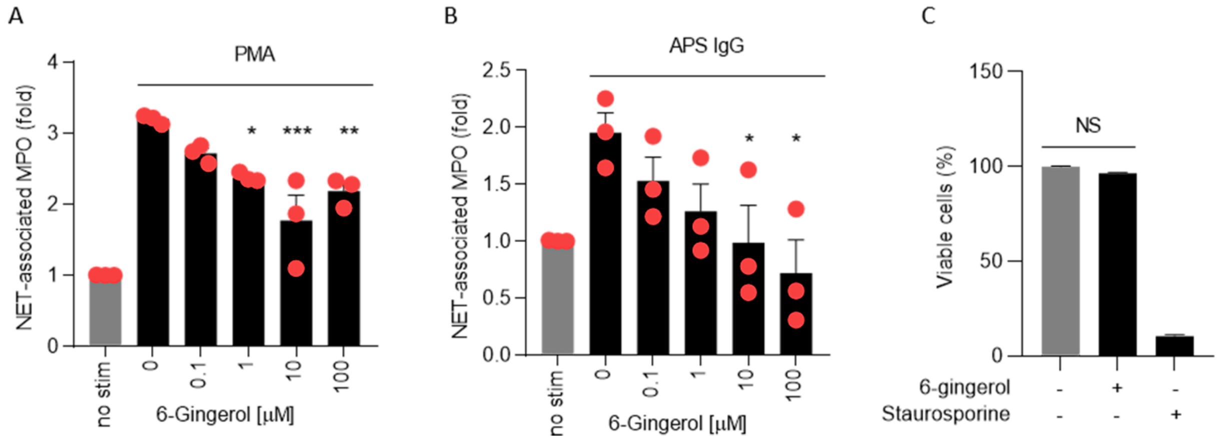

Supplemental Figure 1: *In vitro* efficacy and toxicity of 6-gingerol. Dose response to PMA- (**A**) and APS-mediated (**B**) NETosis upon treatment with 6-gingerol. For viability studies, neutrophils were cultured in the presence or absence of 1 mM 6-gingerol for 3 hours. Trypan blue was used to stain the dead cells, and viable cells were counted thereafter. For all panels, mean and standard error of the mean (SEM) are presented for $n = 3$ independent experiments; *$p < 0.05$, **$p < 0.01$, and ***$p < 0.001$ as compared with the 0-μM gingerol group by one-way ANOVA corrected with Dunnett's test; NS=not significant.

Supplemental Figure 2

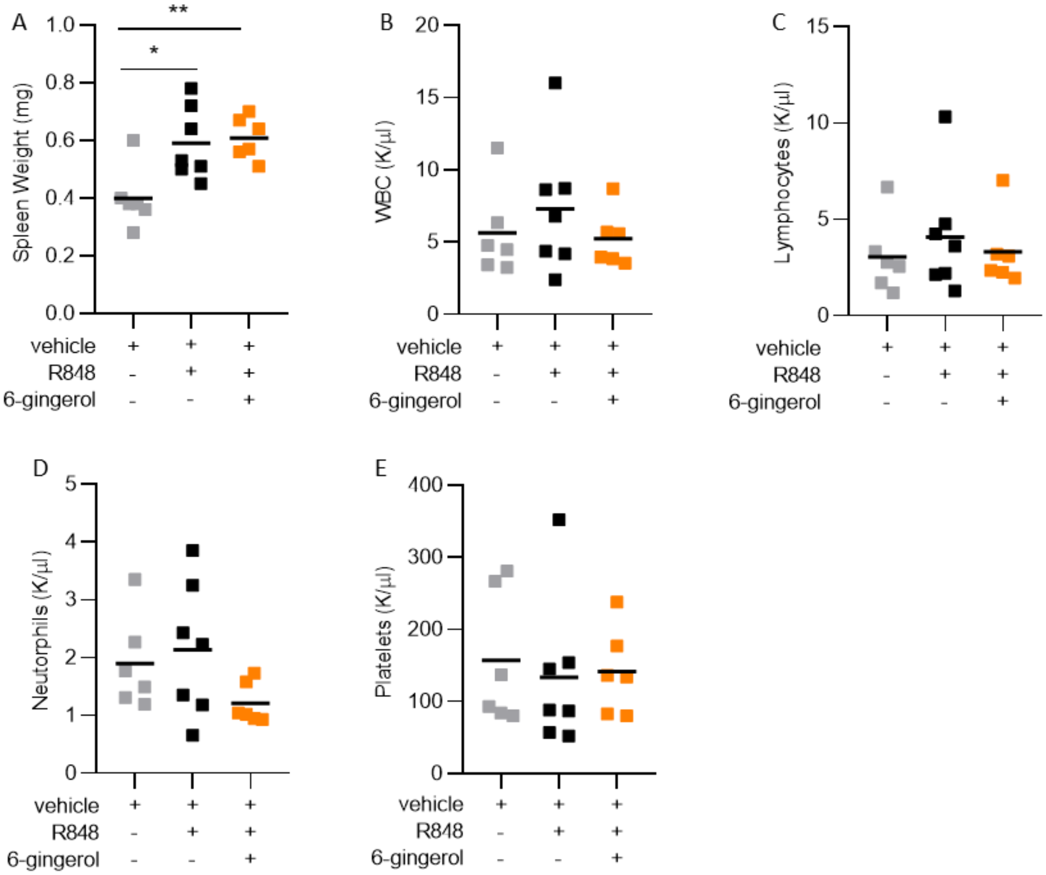

Supplemental Figure 2: BALB/c mice were treated topically with TLR7 agonist (R848) or vehicle DMSO (3x per week) as in Figure 4. Some mice were additionally injected (IP) with 20 mg kg^{-1} of 6-gingerol (3x per week). Six weeks later, various end points were assessed. spleen size (A) was measured. White blood cells (WBC) (B), lymphocytes (C), neutrophils (D), and platelets (E) were quantified in peripheral blood. Mean is presented as a horizontal line; *$p < 0.05$, **$p < 0.01$, by one-way ANOVA Tukey’s multiple comparison.

Supplemental Figure 3

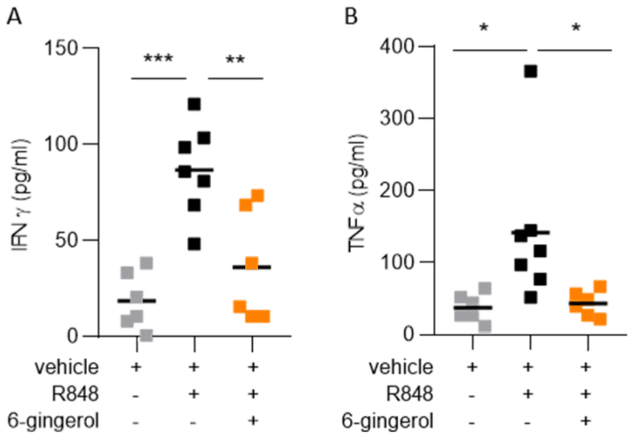

Supplemental Figure 3: 6-gingerol suppresses pro-inflammatory cytokine production in a lupus mouse model. BALB/c mice were treated topically with TLR7 agonist (R848) or vehicle DMSO (3x per week) as in Figure 4. Some mice were additionally injected (IP) with 20 mg kg^{-1} of 6-gingerol (3x per week). Six weeks later, pro-inflammatory cytokines IFN-γ (**A**) and TNF-α (**B**) were measured in serum by ELISA. For all panels, mean is presented as a horizontal line; *$p < 0.05$, **$p < 0.01$, ***$p < 0.001$ as compared with the R-848-alone group by one-way ANOVA corrected with Dunnett's test.

Supplemental Figure 4

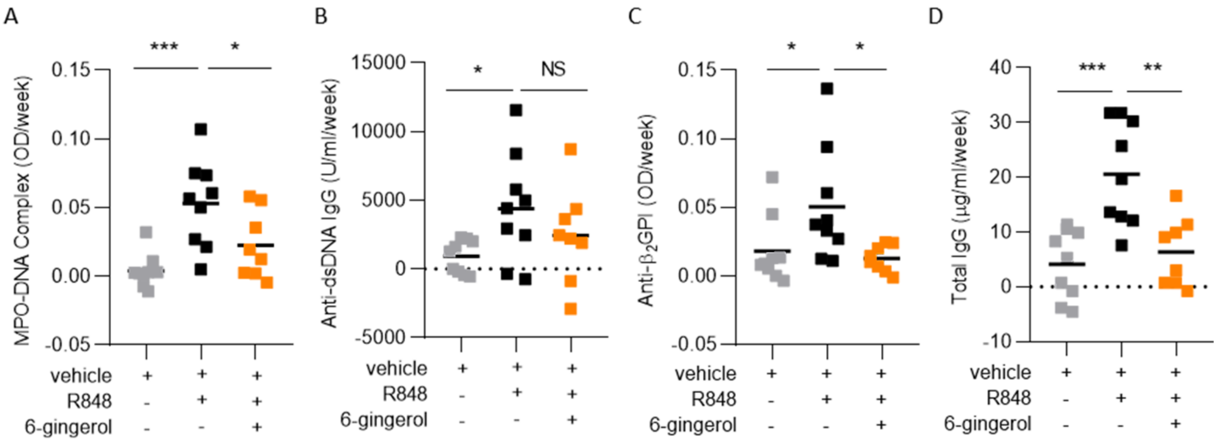

Supplemental Figure 4: Slope of NETs and autoantibodies before and after 6-gingerol treatment in a lupus mouse model. BALB/c mice were treated topically with TLR7 agonist (R848) or vehicle DMSO for 6 weeks (3x per week). Starting at week 4, some mice were additionally injected (IP) with 20 mg kg^{-1} 6-gingerol (3x per week). The change in NET levels (**A**), anti-double stranded DNA (anti-dsDNA) (**B**), anti-beta-2 glycoprotein I (anti-β_2GPI) IgG (**C**), and total IgG (**D**) in serum before and after 6-gingerol treatment by was calculated and the slope of each individual mouse was plotted (**A**). For all panels, mean is presented as a horizontal line; *$p < 0.05$, **$p < 0.01$, ***$p < 0.001$ by paired t test.

Supplemental Figure 5

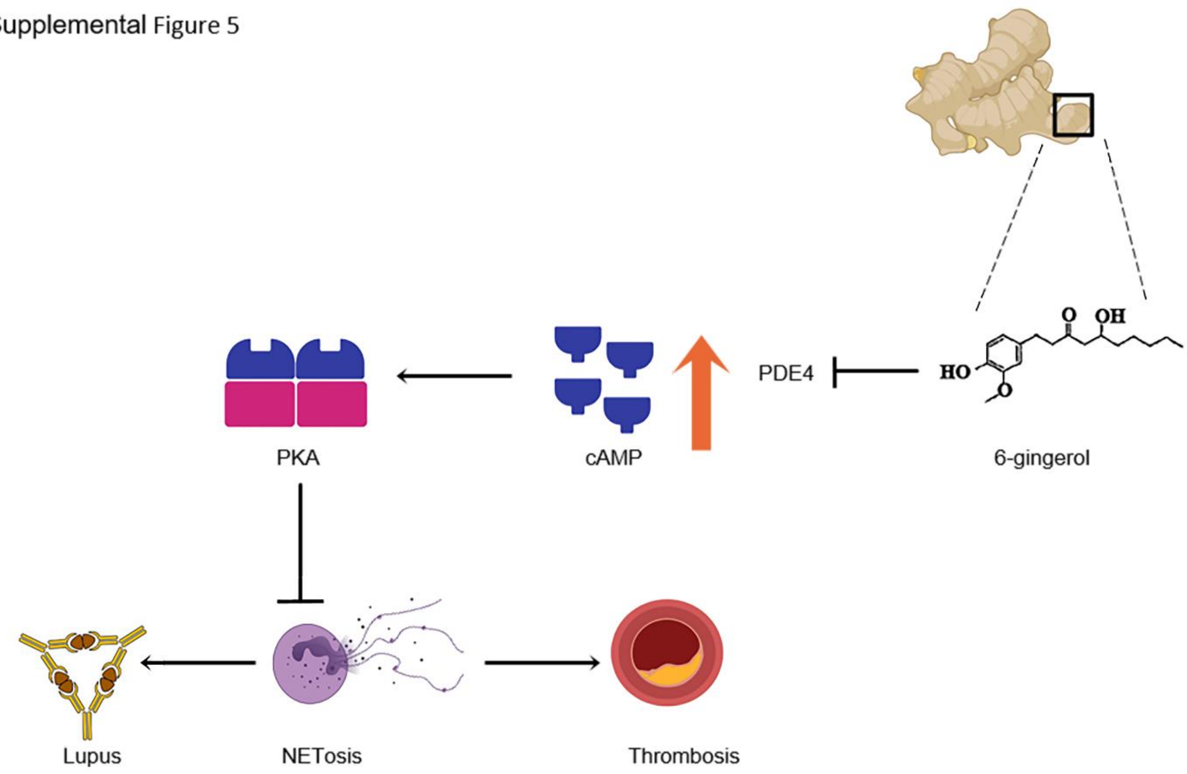

Supplemental Figure 5: Graphical presentation of 6-gingerol anti-neutrophil properties in models of lupus. 6-gingerol inhibits PDE4 thereby increasing intracellular concentrations of cAMP and enhancing PKA activity in neutrophils; thus, suppressing NET release in models of lupus, while also attenuating other disease-relevant activities such as autoantibody formation and large-vein thrombosis.
